# Effect of Forced Physical Activity on the Severity of Experimental Colitis in Normal Weight and Obese Mice. Involvement of Oxidative Stress and Proinflammatory Biomarkers

**DOI:** 10.3390/nu11051127

**Published:** 2019-05-21

**Authors:** Jan Bilski, Agnieszka Mazur-Bialy, Dagmara Wojcik, Marcin Magierowski, Marcin Surmiak, Slawomir Kwiecien, Katarzyna Magierowska, Magdalena Hubalewska-Mazgaj, Zbigniew Sliwowski, Tomasz Brzozowski

**Affiliations:** 1 Department of Ergonomics and Exercise Physiology, Faculty of Health Sciences, Jagiellonian University Medical College, 20 Grzegorzecka Street, 31-531 Cracow, Poland; mpbilski@cyf-kr.edu.pl (J.B.); agnieszka.mazur@uj.edu.pl (A.M.-B.); 2 Department of Physiology, Faculty of Medicine, Jagiellonian University Medical College, 16 Grzegorzecka Street, 31-531 Cracow, Poland; dagmara1.wojcik@uj.edu.pl (D.W.); m.magierowski@uj.edu.pl (M.M.); msurmiak@cm-uj.krakow.pl (M.S.); skwiecien@cm-uj.krakow.pl (S.K.); katarzyna.magierowska@uj.edu.pl (K.M.); madzia_hubalewska@wp.pl (M.H.-M.); AgaZS@poczta.fm (Z.S.)

**Keywords:** forced treadmill exercise, colitis, oxidative stress, high-fat diet, lipid peroxidation, proinflammatory cytokines, hypoxia inducible factor-1alpha

## Abstract

Inflammatory bowel diseases are a heterogeneous group of disorders represented by two major phenotypic forms, Crohn’s disease and ulcerative colitis. Cross talk between adipokines and myokines, as well as changes in intestinal microcirculation, was proposed in pathogenesis of these disorders. C57BL/6 male mice were fed ad libitum for 12 weeks a standard (SD) or high-fat diet (HFD). After the adaptation period, two groups of animals fed SD or HFD were subjected to 6 weeks of the forced treadmill exercise and the experimental colitis was induced in both groups of sedentary and exercising mice fed SD and HFD by intra-colonic administration of 2,4,6-trinitrobenzenesulfonic acid. The disease activity index (DAI), colonic blood flow (CBF), the weight of animals, caloric intake, the mesenteric fad pad, the colonic oxidative stress markers malondialdehyde (MDA), reduced glutathione (GSH), and superoxide dismutase (SOD) activity and intestinal expression and protein content of proinflammatory markers were evaluated. Macroscopic and microscopic colitis in sedentary SD mice was accompanied by a significant fall in CBF and exacerbated in those fed a HFD. The contents of MDA, GSH, and SOD activity were significantly increased in both SD and HFD fed mice with treadmill exercise as compared with sedentary mice. In sedentary HFD mice a significant increase in the intestinal oxidative stress parameters and mucosal expression of IL-1β, TNF-α, IL-17, IFNγ, IL-6, and IL-10 protein were observed and these effects were aggravated in mice subjected to forced treadmill exercise. The mucosal expression of mRNA for TNF-α, IL-1β, iNOS, COX-2, SOD-1, SOD-2, GPx mRNAs, and the hypoxia inducible factor (HIF)-1α protein expression were upregulated in colonic mucosa of treadmill exercising HFD mice with colitis compared with those without exercise. We conclude that forced treadmill running exacerbates the severity of colonic damage in obese mice due to a fall in colonic microcirculation, an increase in oxidative stress, and the rise in expression and activity of proinflammatory biomarkers.

## 1. Introduction

Inflammatory bowel diseases (IBD) such as Crohn’s disease (CD) and ulcerative colitis (UC) are chronic, relapsing, and remitting intestinal disorders of inflammatory conditions of the colon and small intestine that markedly diminish physical functioning and quality of life in patients [1]. For patients in remission or with less active disease, treatment consists of relapse prevention and improvement of quality of life [2]. Physical exercise has been proposed as a potentially useful adjunctive therapy for such IBD patients because it could counteract some IBD-specific complications by improving bone mineral density, muscle mass and strength, immunological response, psychological health, and nutritional status [3]. The beneficial effects of regular moderate exercise may be in part due to the anti-inflammatory effects of myokines such as irisin released by exercising muscles [4,5,6,7]. 

While IBDs etiology is unknown, it is now accepted that a combination of environmental agents, the disturbances in the intestinal microbiome, and a dysfunction of mucosal immune system in genetically susceptible individuals may lead to chronic inflammatory state and the development of either CD or UC [8]. The incidence rates and prevalence of IBD over the last 50 years have increased remarkably in countries that have adapted a “westernized” lifestyle [9,10] possible because of various modifications in dietary habits and decreased physical activity. Even though most of CD patients are underweight, the ratio of intraabdominal fat to total abdominal fat is far greater than in control groups [11]. In CD the hypertrophied mesenteric adipose white tissue (mWAT) could be a major contributor of the increased circulating pro-inflammatory cytokines and plays a role in the pathogenesis and activity of the disease [12,13,14]. Diet-induced obesity by feeding rodents a high-fat diet (HFD) mimics human characteristics of obesity and leads to the increased mesenteric fat deposition, the development of colonic inflammation and the deterioration of experimental colitis [15,16,17]. On the other hand, voluntary exercise has been shown to attenuate the severity of colonic damage in mice fed a HFD by involving the attenuation of the proinflammatory cytokines release and restoration of the plasma levels of protective adiponectin [16].

The anti-inflammatory effects of regular exercise could explain the well-documented beneficial effects of physical activity in several chronic diseases. There is, as confirmed by epidemiological studies, an inverse association between physical activity and markers of low-grade systemic inflammation [18]. However, a prolonged or strenuous exercise may have pro-inflammatory action and leads to increased tissue and plasma levels of pro-inflammatory cytokines. Such exercise can also place a considerable strain on the antioxidant system that leads to the intensification of the oxidative stress causing the dysregulation of inflammatory and neuroendocrinology systems [19]. It was proposed that the effects of training depend largely on the type, intensity and volume of the exercise [20,21]. Animal studies allow comparing exercise programs of different design and intensity and thereby better understanding the mechanisms through which exercise exerts protective effects. The beneficial effects of voluntary exercise on intestinal impairment during colitis is well-proven and was recently thoroughly reviewed [3] but the effect of forced exercise on the course of experimental colitis and intestinal inflammatory parameters has been little studied [22]. On the other hand, some studies have also shown that moderate forced treadmill exercise training can exert an anti-inflammatory effect in the inflamed colon [23,24]. These observations indicate that exercise intensity plays an essential role in the final outcome of either exacerbation or resolution of intestinal changes associated with the voluntary vs. forced exercise applied under experimental conditions. Recently, it has also been shown that a high-fat diet exacerbates experimental colitis and a beneficial effect of moderate exercise was postulated [15,24,25,26,27,28]

Therefore, in the present study we attempted to determine the effect of forced physical activity in mice fed normal standard diet (SD) or HFD on the outcome of the experimental colitis induced by intrarectal administration of 2,4,6-trinitrobenzenesulfonic acid (TNBS). We have chosen the TNBS-induced colitis mice model, because this experimental model is generally thought to resemble Crohn’s disease with mucosal inflammation mediated by a Th1 response with excessive proinflammatory cytokine production and associated with reduced skeletal muscle mass and mWAT hypertrophy [29,30]. We studied the effect of forced treadmill on the disease activity index (DAI), colonic blood flow (CBF) and the colonic expression and concentration of proinflammatory biomarkers in mice fed with different diets. We have also examined the alteration in expression of hypoxia inducible factor (HIF)-1α and anti-inflammatory heme oxygenase (HO)-1 in mice fed with SD and HFD exposed to forced treadmill exercise.

## 2. Materials and Methods

### 2.1. Animals and Diets

The animal protocol was designed to minimize pain or discomfort to the animals. Animal studies were carried out in eighty male C57BL/6J mice kept in a pathogen-free room with free access to water and food and kept in laboratory conditions with 12 h/12h day/night cycles. Mice were fed ad libitum for 12 weeks a regular chow pellets as a standard diet (SD, diet C 1000; Altronim, Lage, Germany) or the high-fat diet (HFD) C 1090-70 obesity-inducing diet with w/70% energy from fat (42% fat) as described previously [16]. The energy values for both diets are: C 1000 (control): 3518.05 kcal/kg and C 1090-70 Obesity: 5495.855 kcal/kg. Besides of 42.0% crude fat, the HFD contained 6.1% crude ash, 4.7% crude fiber, 21.1% crude protein, 21.7% nitrogen free extractives, and 4.4% moisture. The detailed composition of both diets can be found under the link below (http://www.altromin.com/products/special-and-experimental-diets/data-specifications-of-special-diets/#kontroll).

The study was approved by the local Ethical Committee at the Jagiellonian University Medical College in Cracow, Poland (No. 158/2013) and was run in accordance with the Helsinki declaration and with implications for replacement, refinement or reduction (the 3Rs) principle (Decision No.: 22/2016; date: 20 July 2016).

### 2.2. Experimental Design

After the adaptation period, SD and HFD fed mice were randomly assigned into four experimental series, each consisting of 8-10 animals per group: (1) sedentary mice kept on SD, (2) mice maintained on SD and subjected to forced treadmill exercise, (3) sedentary mice fed HFD and (4) mice fed HFD and subjected to forced treadmill exercise. After 12 weeks of HFD (C 1090-70 Obesity diet, Altromin, Germany) and SD (C 1000) feeding, two groups of animals fed SD or HFD were subjected to the forced treadmill exercise to assess the effect of physical activity on the course of experimental colitis. During the exercise sessions, the animals assigned to exercise group were run on a two-lane, 0% incline treadmill (Harvard Apparatus, MA, USA). The speed of the treadmill was precisely controlled because this device is equipped with an endless conveyor-type belt, driven by a DC servomotor with optical encoder. The animals were separated from each other by opaque partitions. The motor drive electronics permits the user to select any speed from 0 up to 100 m per min. In our experiments, the speed of 12 cm/sec for 30 min each day throughout the period of 6 weeks in total has been selected.

At this speed we did not have to use any negative reinforcement, which could additionally contribute to stress. Foam sponges were placed at the back of the treadmill lanes to offer tactile sensation and to prevent injuries to the animals. During the time of experimentation, the sedentary and exercising mice were still maintained on C 1000 standard and C 1090-70 obesity diet, respectively. Sedentary mice were exposed to the treadmill environment for a similar time but did not exercise. After the end of 6 weeks of forced treadmill exercise, the experimental colitis was induced in both groups of sedentary and exercising mice fed SD and HFD by intra-colonic administration of TNBS as described elsewhere [16].

### 2.3. Induction of Colitis

The animals were anaesthetized with isoflurane and experimental colitis in randomly assigned lean fed SD and HFD mice was induced by intracolonic administration of TNBS (Sigma, Slough, United Kingdom) at a dose of 100 μg/g in 40% ethanol. For this purpose, the volume of 175 μL volume of TNBS dissolved in 40% solution of ethanol or an equal volume of 0.9% saline solution was instilled via rectum on day 0. Animals in the control group were administered with 40% ethanol given in a volume of 0.175 µL per mice, corresponding to mice that have been administered TNBS. Following the induction of colitis, animals were housed individually, and daily food intake and body weight were monitored. After the induction of colitis with TNBS, the exercise sessions were not continued. At day 4 post colitis induction, the animals were weighed and anaesthetized and after opening of their abdominal cavity the CBF was determined using the Laser Doppler flowmetry in the areas of colonic mucosa not affected by inflammatory lesions as described before [24,31]. Immediately after the end of CBF determination, animals were killed The DAI and rectal bleeding scores were calculated using a modification of a previously published compounded clinical score. In brief, the DAI consisted of a scoring for diarrhea and lethargy (0–3), whereas rectal bleeding consisted of a visual observation of blood in feces and the perianal area (0–4) [24].

### 2.4. Assessment of Microscopic Changes in the Colonic Mucosa

For histology determination, the gastric tissue sections were excised and fixed in 10% buffered formalin, pH = 7.4. Samples were dehydrated by passing them through a series of alcohols with incremental concentrations, equilibrated in xylene for 10–15 min and embedded in paraffin; paraffin blocks were cut into about 4 μm sections using a microtome. The prepared specimens were stained with hematoxylin/eosin (H&E) and evaluated using a light microscope (AxioVert A1, Carl Zeiss, Oberkochen, Germany). Digital documentation of histological slides was obtained using above mentioned microscope equipped with automatic scanning table and ZEN Pro 2.3 software (Carl Zeiss, Oberkochen, Germany). For histology assessment formalin-fixed full-thickness samples were serially cross-sectioned (10 μm-thick). Sections were scored using histomorphological score for intestinal inflammation in mice proposed by Erden et al. [32] 1: mild mucosal inflammatory cell infiltrates with intact epithelium, 2: inflammatory cell infiltrates into mucosa and submucosa with undamaged epithelium, 3: mucosal infiltrates with focal ulceration; 4: inflammatory cell infiltrates in mucosa and submucosa and focal ulceration, 5: moderate inflammatory cell infiltration into mucosa and submucosa with extensive ulcerations; 6: transmural inflammation and extensive ulceration.

### 2.5. Determination of Lipid Peroxidation

To determine malondialdehyde (MDA) and 4-hydroxynonenal (4-HNE) tissue concentration, the colorimetric assay for lipid peroxidation (Bioxytech LPO-586, Oxis, Portland, USA) was used. About 100 mg of colon was excised from mice, tissue being transferred to the vial containing 1 mL of 20 mM PBS (pH = 7.4) and 10 μL of 0.5 M of butylated hydroxytoluene in acetonitrile in order to prevent sample oxidation. Samples were subsequently mechanically homogenized for 15 s. The homogenates were centrifuged for 10 min (3000 *g* at 4 °C). The obtained clear supernatant was stored at −80 °C prior to testing. The colorimetric assay used to determine MDA concentration in gastric mucosa is based on the reaction of a chromogenic reagent (N-methyl-2-phenylindole) with MDA and 4-HNE at 45 °C, which yields a stable chromophore with maximal absorbance at 586 nm, analyzed with a microplate reader (Tecan Sunrise, Männedorf, Switzerland). Results were expressed as nanomoles per gram of colonic tissue (nmol/g).

### 2.6. Measurement of Reduced Glutathione (GSH) Content

For the measurement of the concentration of reduced form of glutathione (GSH), the colorimetric assay (Bioxytech, GSH-400, Oxis, Portland, OR, USA) was used. The method is based on a chemical reaction in which a chromophoric thione with a maximal absorbance wavelength at 400 nm is obtained. The colonic sample of about 100 mg was collected and homogenized in ice-cold 5% metaphosphoric acid solution in order to evoke protein precipitation. The homogenates were centrifuged for 10 min (3000 *g* at 4 °C). The upper clear aqueous layer was collected and assayed within 1 hour. The level of reduced glutathione was measured with maximal absorbance at 400 nm by a microplate reader (Tecan Sunrise, Männedorf, Switzerland). Results were expressed as micromoles per gram of tissue (μmol/g).

### 2.7. Determination of Superoxide Dismutase (SOD) Activity

To determine the activity of SOD, a sample of colonic mucosa was collected and homogenized in cold 20 mM HEPES buffer (pH = 7.2) containing 1 mM EGTA, 210 mM mannitol, and 70 mM sucrose per gram of colonic tissue and centrifuged for 5 minutes (1500 g at 4 °C). The supernatant was collected and assayed immediately using the colorimetric assay for assessment of SOD activity (Cayman Chemical, MI, USA). Cayman’s Superoxide Dismutase Assay Kit utilizes a tetrazolium salt for detection of superoxide radicals generated by xanthine oxidase and hypoxanthine. One unit of SOD is defined as the amount of enzyme needed to exhibit 50% dismutation of the superoxide radical. The absorbance was measured by microplate reader (Tecan Sunrise, Männedorf, Switzerland) at 450 nm and the results were expressed as units per gram of colonic tissue (U/g).

### 2.8. Luminex Microbeads Fluorescent Assays

Determination of interferon (IFN)-γ, interleukin (IL)-6, IL-10, IL-17, IL-1β, tumor necrosis factor (TNF)-α levels in colonic tissue was performed using Luminex microbeads fluorescent assays (Bio-Plex Pro™ Mouse Cytokine Th17 Panel A 6-Plex #M6000007NY) and Luminex 200 system (Luminex Corp., Austin, TX, USA) [31,33]. Results were calculated from calibration curves and expressed in pg/mg of tissue.

### 2.9. Gene Expression in the Mouse Colonic Mucosa Determined by the Real Time Polymerase Chain Reaction (Real-Time PCR)

The mRNA expression for TNF-α, IL-1β, cyclooxygenase-2 (COX-2), inducible nitric oxide synthase (iNOS), SOD-1, SOD-2 as well as glutathione peroxidase (GPx-1) was determined in colonic mucosa, which was immediately snap frozen after collection in liquid nitrogen and stored at −80 °C until analysis. The total RNA was extracted from samples using RNeasy Mini Kit (Qiagen, 74134, Hilden, Germany) according to the manufacturer’s protocol. The RNA concentration and purity was measured in NanoDrop 2000 spectrophotometer (Thermo Fisher Scientific, Wilmington, DE, USA). Reverse transcription was performed using the High Capacity RNA-to-cDNA Kit (4387406; ThermoFisher; Foster City, CA, USA) based on the manufacturer’s instruction. Real-time PCR was performed using Power SYBR™ Green PCR Master Mix (4367659; Applied Biosystems; ThermoFisher; Foster City, CA, USA) in Step One Plus thermocycler (Applied Biosystems) with a β-actin as a housekeeping gene. The sequences of primers used in real time PCR are listed in Table 1. Relative gene expression was calculated based on the obtained Ct values using the 2−ΔΔCt method.

### 2.10. Colonic Expression of HO-1 and HIF-1α Proteins Assessed by Western Blot

Proteins were isolated from colonic mucosa using appropriate homogenization buffer (50 mM Tris 7.5 pH, 130 mM NaCl, protease inhibitor cocktail (SIGMAFAST Protease Inhibitor Cocktail Tablets, Sigma Aldrich, Schnelldorf, Germany)) and 1% NP-40. Expressions for HO-1, HIF-1α and β-actin as loading control were determined in colonic mucosa using western blot, as described in our previous studies [33]. Rabbit monoclonal anti-HO-1 (ab68477, Abcam, Cambridge, UK) in dilution of 1:2000, rabbit polyclonal anti-HIF-1α (14179, Cell Signalling Technology, Danvers, MA, USA) in dilution of 1:1000, mouse monoclonal anti-β-actin (3700S, Cell Signalling Technology) in dilution of 1:1000 were used as primary antibodies. Protein expression was visualized using HRP-linked secondary goat anti-rabbit IgG H&L antibody (ab97051, Abcam) in dilution of 1:2000 or anti-mouse IgG antibody (7076P2, Cell Signalling Technology) in dilution of 1:2000 where appropriate. Anti-HIF-1α antibody was diluted in 5% bovine serum albumin and all other primary and secondary antibodies were diluted in 5% non-fat milk. Chemiluminescence was developed using WesternSure^®^ ECL Substrate (LI-COR, NE, USA) or WesternBright Quantum (Advansta, Menlo Park, CA, USA) and was measured using C-DiGit^®^ Blot Scanner (LI-COR). The intensity of bands was determined and analyzed using Image Studio 4.0 software (LI-COR). The expression of each protein of interest was normalized to the expression of β-actin as loading control [33].

### 2.11. Statistical Analysis

Results are expressed as means ± SEM. The data was processed by the statistical analysis software SPSS version 16.0 (SPSS Inc., Chicago, IL, USA). Statistical analysis was done using two-way ANOVA test with Tukey post hoc test where appropriate. Differences of *p* < 0.05 were considered significant.

## 3. Results

### 3.1. The Effect of Forced Treadmill Exercise on DAI Activity, CBF, and Macroscopic and Microscopic Appearance of Colonic Mucosa in Mice with Colitis

**Figure 1:**
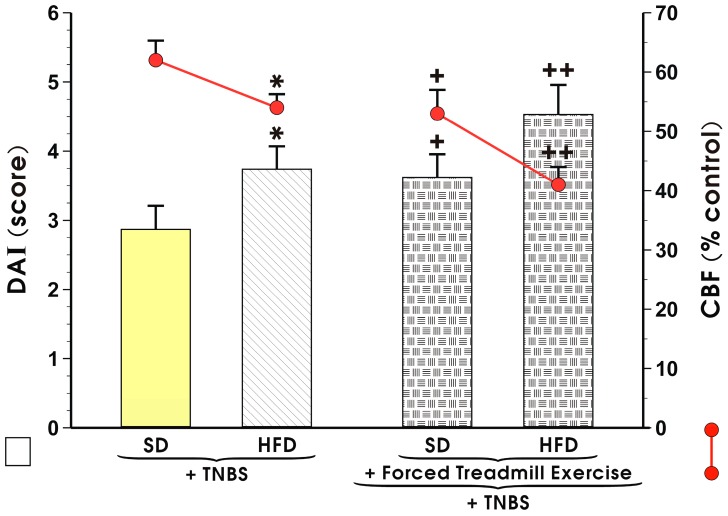
Effect of exercise on the disease activity index (DAI) and the accompanying alterations in colonic blood flow (CBF) presented as red line in this Figure in sedentary mice with TNBS-induced colitis fed standard diet (SD) or high-fat diet (HFD) with or without forced treadmill exercise. Results are Mean ± S.E.M. of 8 animals per each group. An asterisk indicates a significant change (*p* < 0.05) as compared to respective values in sedentary mice fed SD. Cross indicates a significant change in (*p* < 0.05) as compared to the respective values obtained in animals fed SD not subjected to forced exercise. Double crosses indicate a significant change in (*p* < 0.05) as compared to the respective values obtained in animals fed SD with forced exercise.

Figure 1 shows the alteration in the mucosal DAI and changes in CBF in sedentary mice with TNBS colitis fed a SD and those fed a HFD with or without forced exercise. The DAI was markedly increased and CBF was substantially reduced in the HFD mice without exercise as compared to the respective values recorded in those maintained on a SD (*p* < 0.05) (Figure 1). When the mice fed a SD or HFD were subjected to forced exercise and administered with TNBS, further significant increase in DAI and a significant fall in CBF was observed as compared to those maintained under sedentary condition and fed SD or HFD (*p* < 0.05) (Figure 1). 

**Figure 2:**
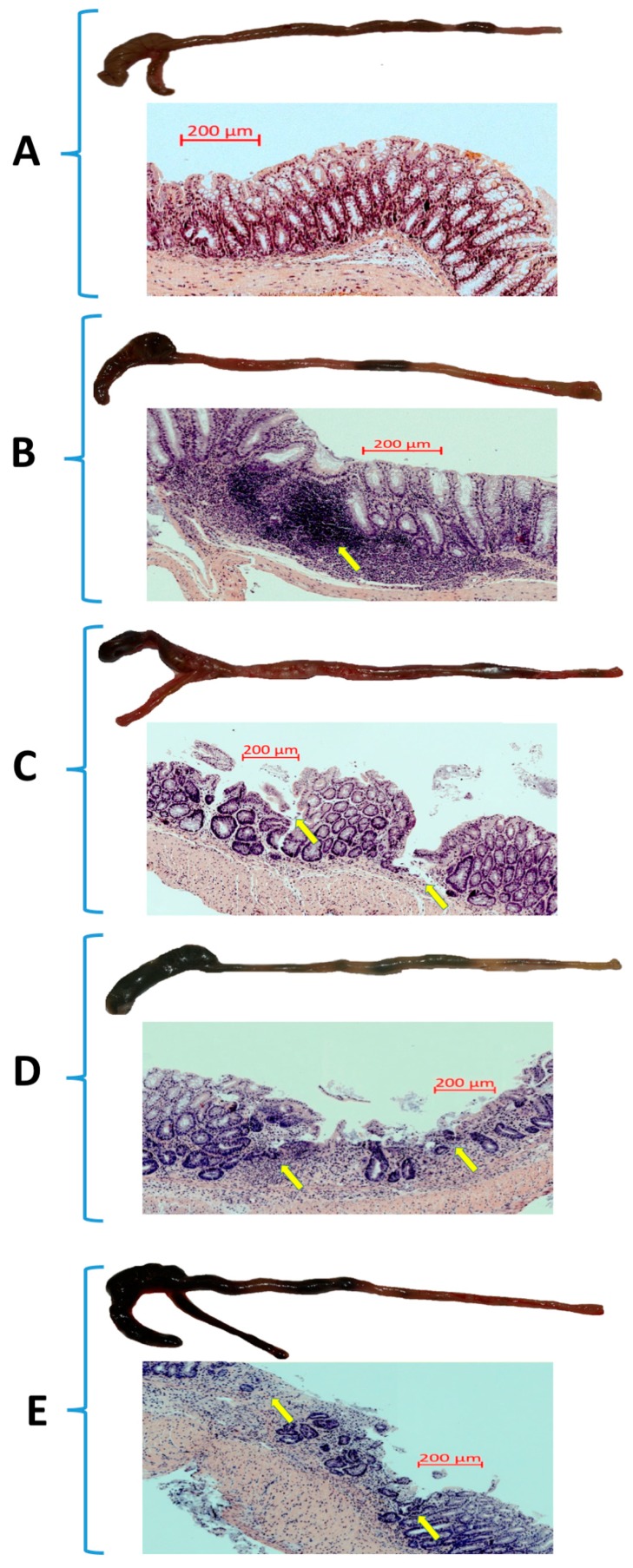

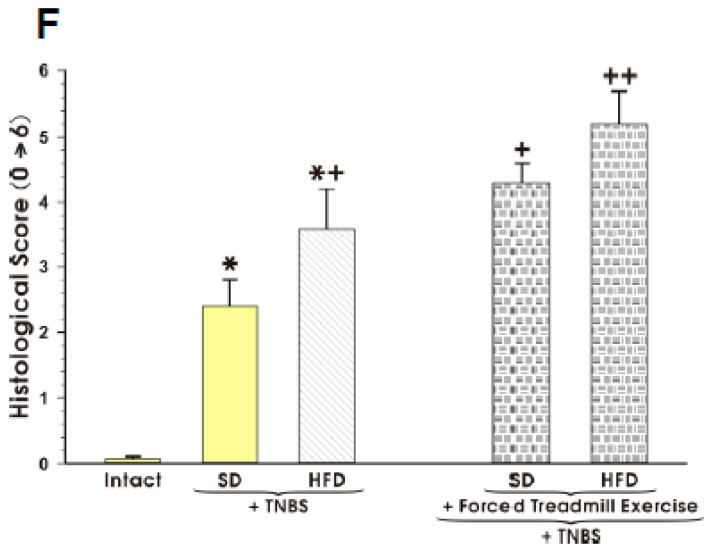
The representative gross (upper panel) and microscopic (lower panel) appearance of colon from intact (**A**), sedentary mouse with TNBS colitis fed standard diet (SD) (**B**), sedentary mouse fed high-fat diet (HFD) with colitis (**C**), sedentary mouse fed SD exposed to forced exercise training (**D**), or mouse fed HFD with colitis exposed to forced treadmill exercise (**E**) and the quantitative histology scores in non-exercising and exercising mice with colitis (**F**). In mice fed SD or HFD with colitis subjected to forced exercise, the enterocyte architecture is distorted revealing destroyed crypts, extended ulcerations and transmural inflammatory cell infiltrates (**D**,**E**) (arrows) as compared with mice fed either with SD or HFD but not exposed to forced treadmill exercise (**B**,**C**). These values of histopathological score were significantly increased in non-exercising and exercising mice fed HFD. An asterisk indicates a significant change (*p* < 0.05) vs. intact colonic mucosa. An asterisk and cross indicate a significant change (*p* < 0.05) as compared to sedentary mice fed SD. Cross indicates a significant change (*p* < 0.05) as compared with respective values in sedentary mice fed SD. Double crosses indicate a significant change (*p* < 0.05) as compared with respective values in exercising mice fed SD.

The representative gross macroscopic and microscopic appearances of the colon of mice fed both SD and HFD with or without exercise are presented in Figure 2A–F. The macroscopic appearance of intact colon (Figure 2A) and that of sedentary mice with TNBS colitis fed SD or HFD are presented in Figure 2B,C, respectively, while colons of mice with TNBS colitis fed SD or HFD and subjected to forced treadmill exercise are shown in Figure 2D,E, respectively. The intact colon from the sedentary mouse fed a SD showed the normal physiological architecture without any macroscopically or microscopically detectable signs for intestinal injury (Figure 2A). In the sedentary mice fed a SD with colitis the clearly visible bloody enema reflecting intestinal mucosal damage was observed (Figure 2B). Microscopically, the intestinal mucosal injury was evident in the sedentary mouse fed a SD and administered with TNBS as reflected by the necrosis of the epithelium and focal lesions of the colonic mucosa (Figure 2B). These macroscopic and microscopic changes were more severe with deeper mucosal lesions in the sedentary mice with colitis fed a HFD as compared to those fed on SD without forced exercise (Figure 2C). When mice fed either SD or HFD were subjected to forced treadmill exercise, the exacerbation of the bloody appearance of the colon was observed by gross inspection reflecting much worse outcome of inflammatory reaction as compared to the sedentary mice fed a SD or HFD (Figure 2D,E). These microscopic alternations accompanied by loss of typical architecture and severe neutrophil infiltration of the colonic mucosa were augmented in mouse fed SD or HFD subjected to treadmill exercise (Figure 2D,E). The severity of colonic inflammation in exercising mice compared with sedentary mice as manifested by greater colonic structure disorganization, presence of ulcerations and inflammatory cell infiltration was confirmed by quantitative histology (Figure 2F).

### 3.2. Energy Intake, Body Weight, and Visceral Adiposity in Mice Fed a SD or HFD with or without Forced Treadmill Exercise.

**Figure 3:**
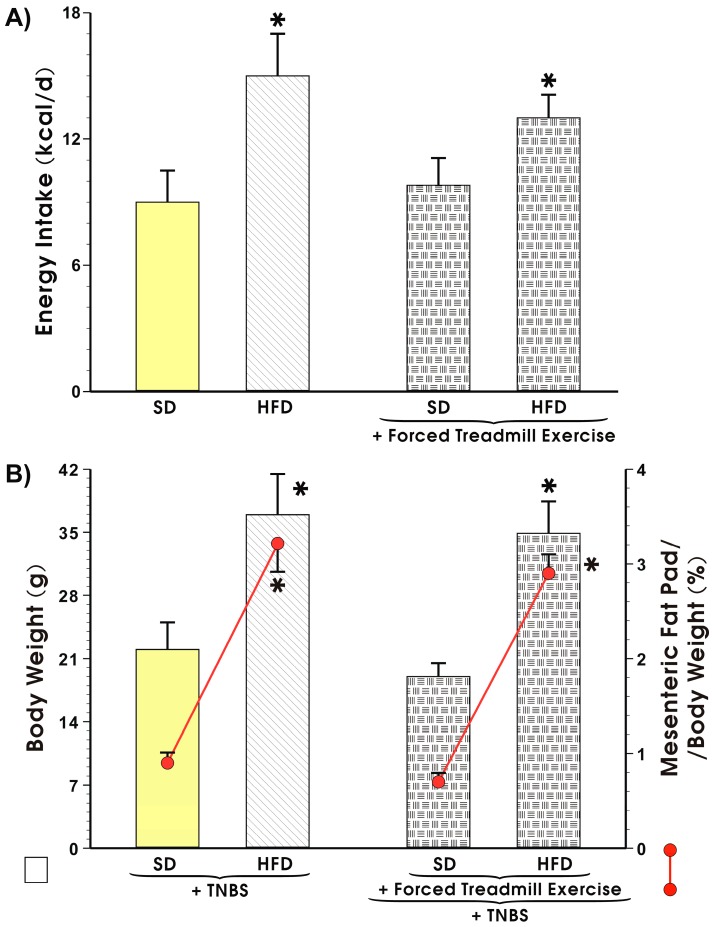
Effect of exercise on the energy intake (**A**), weight gain, and mesenteric fad pad (**B**) depicted as red line, in sedentary mice with TNBS-induced colitis fed SD or HFD with or without forced treadmill exercise. Results are Mean ± S.E.M. of 8 animals per each group. An asterisk indicates a significant change (*p* < 0.05) as compared to respective values in sedentary mice fed SD.

**Figure 4:**
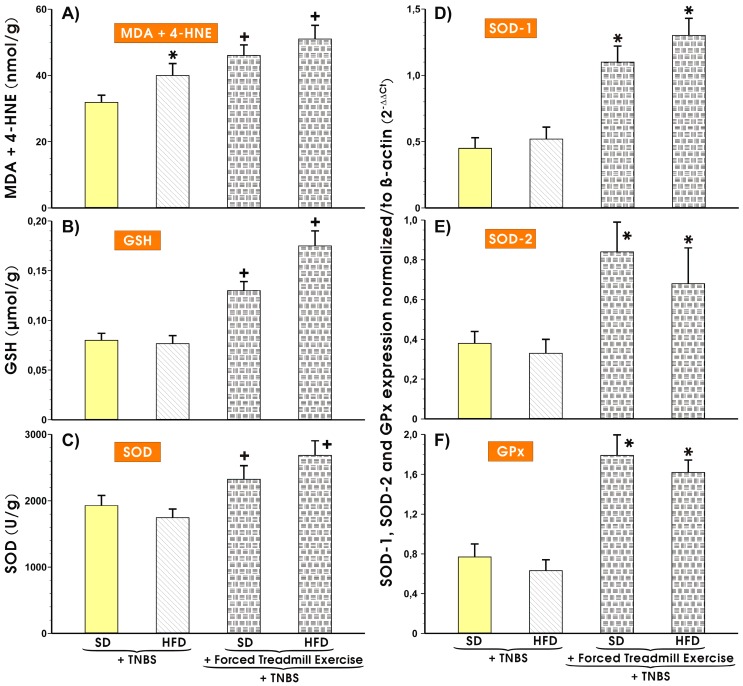
The colonic mucosal content of malondialdehyde (MDA) and 4-hydroxynonenal (4-HNE) (**A**), the reduced glutathione (GSH) (**B**), the activity of superoxide dismutase (SOD) (**C**) and the results of mRNA expression of SOD-1 (**D**), SOD-2 (**E**), and GPx (**F**) in colonic mucosa of sedentary mice with colitis fed SD or HFD with or without forced treadmill exercise. Results are Mean ± S.E.M. of 6 animals per each experimental group. An asterisk indicates a significant change (*p* < 0.05) as compared to respective values obtained in sedentary mice fed SD or HFD. Cross indicates a significant change (*p* < 0.05) as compared with respective values in sedentary mice fed SD or HFD.

The values of energy intake expressed in kcal/day in the sedentary mice fed SD or those fed a HFD, the mice body weight and the weight of mesenteric fat depots expressed as a percentage in the sedentary or exercising mice with or without TNBS colitis fed a SD or HFD are presented in Figure 3A,B, respectively. The energy intake was not significantly different between the sedentary and the exercising mice fed a SD but it was significantly increased in the animals fed a HFD as compared to the value of energy intake obtained in the mice fed a SD (*p* < 0.05, Figure 3A). The body weight in the HFD mice with TNBS colitis was significantly increased as compared to the sedentary animals with TNBS colitis fed SD (Figure 3B). The relative weight of the mesenteric fat depots expressed as percentage was also significantly increased in the HFD fed sedentary mice as compared to the sedentary SD mice with colitis (*p* < 0.05, Figure 3B). In mice fed HFD with colitis and exposed to forced treadmill exercise, the body weight and the mesenteric fat depots were significantly increased as compared with the exercising SD mice with colitis (*p* < 0.05, Figure 4). 

### 3.3. Effects of Forced Treadmill Exercise on the Mucosal Colonic Content of MDA plus 4-HNE, GSH, and SOD Activity and the mRNA Expression of SOD-1, SOD-2, and GPx-1 in Mice with Experimental Colitis

**Figure 5:**
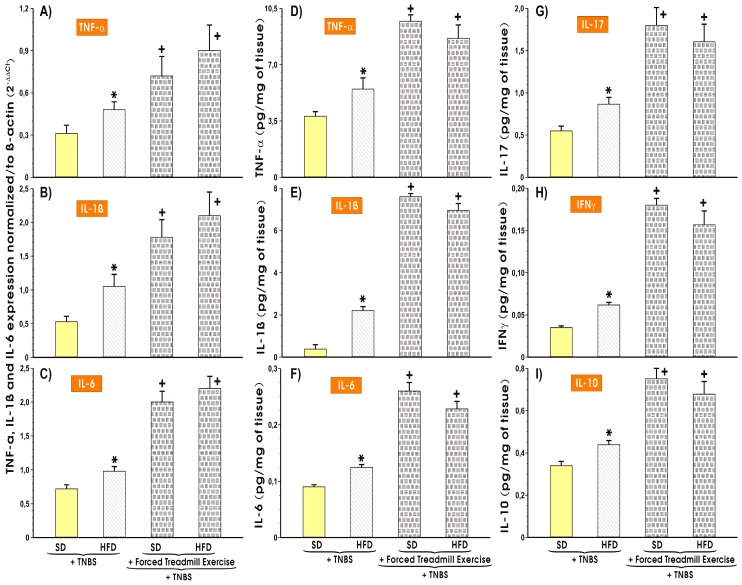
The effect of forced treadmill exercise on the mRNA expression of TNF-α (**A**), IL-1β (**B**), and IL-6 (**C**) and the colonic mucosal tissue levels of IL-1β (**D**), IL-17 (**E**), IL-6 (**F**), TNF-α (**G**), IFNγ (**H**), and IL-10 (**I**) in mice with TNBS-induced colitis fed SD or HFD with or without forced treadmill exercise. Results are Mean ± S.E.M. of 6 animals per each experimental group. Asterisk indicates a significant change (*p* < 0.05) as compared with respective values in sedentary mice fed SD with colitis. Cross indicates a significant change (*p* < 0.05) as compared with respective values in animals fed SD or HFD but not subjected to forced exercise sessions.

Results presented in Figure 4A show that the colonic mucosal MDA and 4-HNE concentration was markedly increased in sedentary mice fed a HFD as compared with those fed SD and administered with TNBS. In SD fed mice subjected to forced exercise a significant increase in the mucosal content of MDA was observed over that measured in sedentary mice on SD (*p* < 0.05) (Figure 4A). The tissue level of MDA was significantly increased in treadmill exercising mice fed HFD as compared to the respective values in sedentary mice with colitis but without exercise (*p* < 0.05) (Figure 4A). In sedentary HFD fed mice with colitis, the GSH content and SOD activity was not significantly different from those recorded in sedentary mice fed SD with colitis but without forced exercise (Figure 4B,C). In contrast, the colonic mucosal contents of GSH and SOD were substantially increased in the colonic mucosa of mice subjected to forced treadmill exercise fed either SD and HFD and administered with TNBS as compared with the respective values of GSH and SOD in sedentary mice with experimental colitis fed SD or HFD (*p* < 0.05) (Figure 4B,C). Figure 5D–F (right panel) shows the results of mRNA expression for SOD-1, SOD-2, and GPx-1 in the colonic mucosa of sedentary or exercising mice with TNBS-induced colitis fed a SD or HFD. The expression of mRNA for SOD-1, SOD-2, GPx, and HO-1 was not significantly altered in colonic mucosa of sedentary mice fed HFD compared with those in animals fed SD. In contrast, the expression of mRNA for SOD-1, SOD-2, and GPx mRNA, was markedly increased in colonic mucosa of mice subjected to forced treadmill exercise as compared to sedentary mice fed both diets (*p* < 0.05) (Figure 4D–F).

### 3.4. The Alterations in the Colonic Expression and Changes in Colonic Mucosal Content of Proinflammatory and Oxidative Biomarkers in Mice with TNBS-Induced Colitis fed SD or HFD with or without Forced Treadmill Exercise 

Figure 5A–C shows the alterations in the mRNA expression of proinflammatory biomarkers TNF-α, IL-1β and IL-6 in the colonic mucosa of sedentary or treadmill exercising mice fed a SD or HFD with colitis and the changes in the colonic mucosal tissue content of proinflammatory (TNF-α, IL 1β, IL-6, IL-17, and INFγ) and anti-inflammatory cytokine IL-10 in mice with TNBS colitis fed SD or HFD with or without forced treadmill exercise (Figure 5D–I). The mRNA expression of IL-1β, TNF-α, and IL-6 mRNA was substantially increased in the colonic mucosa excised from the sedentary animals fed HFD vs. the sedentary mice fed SD (*p* < 0.05) (Figure 5A–C). The forced treadmill exercise markedly increased the IL-1β-, TNF-α-, and IL-6 mRNA expression in colonic mucosa compared with respective semi-quantitative values obtained in sedentary mice fed SD (*p* < 0.05) (Figure 5A–C). The sedentary mice fed HFD with experimental colitis presented significantly higher colonic tissue content of TNF-α, IL 1β, IL-6, IL-17, INFγ, and IL-10 as compared with the respective values of these cytokines recorded in sedentary mice fed SD (*p* < 0.05) (Figure 5 D–I). The cytokine concentrations were significantly enhanced in mice with TNBS colitis fed SD or HFD and subjected to forced treadmill exercise (*p* < 0.05) (Figure 5D–I).

### 3.5. Effect of Forced Treadmill Exercise on mRNA Expression of COX-2, iNOS and Protein Expression of HO-1 and HIF-1α in Mice with Experimental Colitis Fed SD or HFD

**Figure 6:**
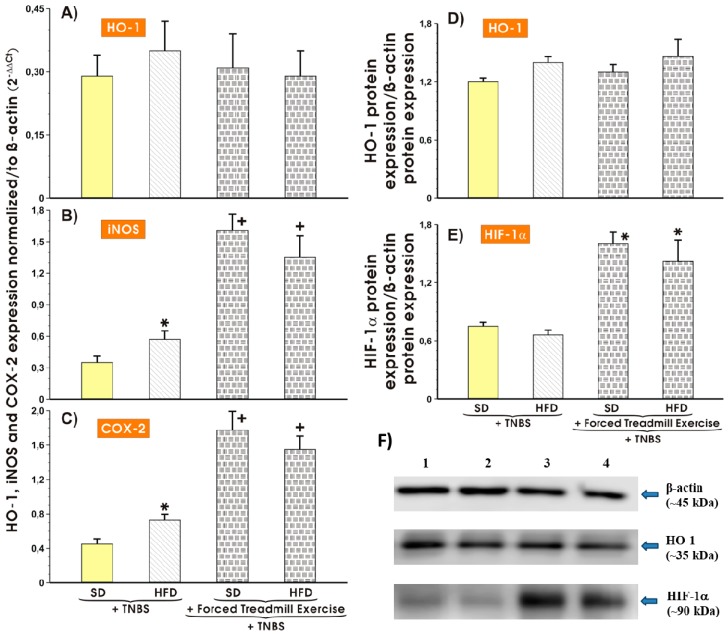
The effect of forced treadmill exercise on the mRNA expression of HO-1 (**A**), iNOS (**B**), and COX-2 (**C**) and the protein expression of β-actin, HO-1, and HIF-1α (**D**–**F**) analyzed in colonic mucosa of mice with colitis fed SD or HFD with or without forced treadmill exercise. Results are mean ± S.E.M. of 6 animals per each experimental group. Asterisk indicates a significant change (*p* < 0.05) as compared with respective values in sedentary mice fed SD. Cross indicates a significant change (*p* < 0.05) as compared with respective values in sedentary mice fed SD or HFD without treadmill exercise.

Figure 6A–F shows the results of mRNA expression of proinflammatory biomarkers HO-1 (A), iNOS (B), and COX-2 (C) and the protein expression of HO-1 and HIF-1α in the colonic mucosa of sedentary or treadmill exercising mice fed either SD or HFD with TNBS-induced colitis. The mRNA expression of HO-1 mRNA remained unchanged in colonic mucosa of mice fed both diets (Figure 6 A) but the expression of iNOS or COX-2 mRNA was substantially increased in the colonic mucosa excised from the sedentary animals fed HFD vs. sedentary mice fed SD (*p* < 0.05) (Figure 6A–C). The colonic expression of iNOS and COX-2 mRNA was further markedly elevated in mice with colitis fed SD and HFD and subjected to forced exercise (*p* < 0.05) (Figure 6B,C). As shown in Figure 6D, the protein expression of HO-1 was not significantly altered in mice fed either SD or HFD with or without forced exercise. In contrast, the significant increase in the protein expression of HIF-1α was observed mice fed SD or HFD and subjected to forced treadmill exercise as compared with the corresponding values obtained in sedentary mice with colitis fed either SD or HFD (*p* < 0.05) (Figure 6E,F).

## 4. Discussion

This study presents the evidence that forced moderate treadmill exercise exacerbated the experimental colitis in HFD fed mice but also to some extent in mice fed SD. This effect was accompanied by the elevation of inflammatory response, the mucosal severity of colitis and an increase in gene and protein expression of proinflammatory biomarkers and colonic oxidative stress as reflected by the rise in MDA content referred to lipid peroxidation. Furthermore, this aggravation of colitis in mice subjected to forced treadmill exercise was linked with the increase in the mucosal expression of proinflammatory mediators and cytokines in the colonic mucosa. These results are in agreement with the data published by Cook et al. [22] who used mice fed SD and exposed them to forced exercise. In their study, the forced treadmill exercise running exacerbated DSS-induced colitis in mice and led to excessive diarrhea episodes with increased animals’ mortality. In contrast, the voluntary wheel running may exert a potent beneficial therapeutic effect on experimental colitis [22]. Such effect has also been confirmed in other experimental studies using mice model [15,16,29]. We have documented in our present study that the intensity of exercise plays an important role in severity and healing rate of experimental colitis in mice fed different diets including HFD. Cook et al. [22] have suggested that the reason for this opposite effect of the voluntary vs. forced exercise is due to the specificity of the forced exercise, which could be perceived as a chronic stressor for these species in contrast with that exerted by voluntary physical activity with moderate intensity. This hypothesis was supported by their observation [22] that mice forced to treadmill exercise, presented adrenal hypertrophy and thymic atrophy. Further support for this idea comes in from the observation that chronic intermittent exposure to a psychosocial stressor before the induction of acute DSS-colitis in mice resulted in adrenal insufficiency and apparently had increased colonic inflammation [34]. Moreover, the mild restraint stress has been found to increase the inflammatory response when TNBS was administered [35]. Stress may predispose the organism to intestinal damage by increasing intestinal permeability and promoting bacterial translocation or by affecting the balance between mucosal oxidant and antioxidant mechanisms [36].

Recently, we have shown accelerated healing of colitis in rats fed with HFD and forced to moderate treadmill running exercise and this effect was accompanied by a downregulation of the expression of inflammatory factors in colonic mucosa [24]. The reason for this discrepancy between results obtained from exercising rats and mice, could be attributed to species difference or exercise intensity because the exercise intensity evidently varied between these animals in both studies. The speed of 20 m/min for 30 min selected in our previous study [24], corresponded to approximately 45–50 VO_2_max [27]. In contrast, speed of 12 cm/s of treadmill in present study were equal to 60–65 VO_2_max [27,28]. This clearly indicates that the exercise applied to mice in present study has a higher intensity then that selected for rats in our previous work [24]. The similar forced treadmill exercise protocols in mice resulted in reduction of adipose tissue mass and systemic inflammation in response to HFD [26,28,36,37,38,39] confirming that the selection of these variables of exercise intensity, namely 12 cm/sec for 30 min each day for 6 weeks can exert considerable physiological effects. We have used HFD fed rats with the increased adiposity leading to the abundance of mesenteric fat pad, and this could serve explanatory for such difference between rats and mice [24]. Interestingly, the colonic inflammation in mice fed a HFD with TNBS-induced colitis was exacerbated and accompanied by an increase in colonic expression of mRNA for inflammatory factors despite smaller intensity of forced exercise than that proposed by study published before [22]. Furthermore, the differences between mice and rats in the species response to psychological stress have been already reported [40,41]. Further studies are needed to test the effect of even lower forced exercise intensity on changes of colonic inflammatory factors. Psychological stress accompanying forced exercise can explain, at least in part, the detrimental effects of forced endurance exercise on the outcome of colitis observed in our study. Indeed, we have clearly demonstrated that the markers of the oxidative stress including MDA content and SOD activity were enhanced in mice fed HFD and subjected to forced exercise as compared with sedentary mice fed SD. However, in the study by Saxena et al. [23], forced treadmill decreased the inflammatory response in the adiponectin knock-out mice with DSS-induced colitis. Thus, the further studies on various intensities of physical activity should shed more light into the understanding of beneficial or deleterious effect depending on intensity of different bouts of exercise, on healing of colonic inflammation in DSS mice model. From the clinical point of view, the effect of moderate exercise applied after colitis had been initially created and then followed by bouts of exercise, will be of great interest. However, this experimental model of TNBS colitis has limitations, which makes this study not feasible since the development of inflammatory intestinal changes in this model is additionally associated with skeletal muscles atrophy. Thus, we assumed that mice at the stage of developing colitis would avoid physical activity similarly to IBD patients who can well tolerate moderate exercise when they are in a quiescent (remission) state of this disease but not when they suffer from an active state of disease. 

Although regular physical activity could be beneficial in the prevention and the management of chronic diseases, including disorders of gastrointestinal tract, the intensive exercise may affect and even exacerbate other existing medical conditions [42,43]. It is also known that intensive exercise such as long distance running and triathlon could evoke the nausea, heartburn, diarrhea, or even gastrointestinal bleeding. Marathon runners suffer from ‘runner’s ischemic colitis’, involving bloody diarrhea, fatigue, and fever [44,45,46,47]. The prevalence of such symptoms is quite high (35–50%), particularly in female runners [43,44,45]. These symptoms could result from prolonged intestinal hypo-perfusion, ischemia, and loss of barrier integrity, but the precise etiology of these disturbances in endurance athletes is still not fully understood. The blood flow to the GI tract and liver during long-term intensive exercise is substantially reduced as the prolonged exercise leads to the blood redistribution from the gut to the exercising muscles [48,49]. Such profound ischemia could lead to mucosal damage, increased intestinal permeability, bacterial translocation through “leaky gut” and elevated circulatory levels of lipopolysaccharides (LPS) [43,50,51]. Such phenomenon is observed after exhaustive exercise [52,53]. LPS could be responsible, for down-stream activation of NF-κB and the increased pro-inflammatory cytokines release frequently observed in these subjects [53,54]. This inflammatory response can derive from the oxidative ischemic stress associated with reperfusion [43,55,56]. Intensive exercise is also associated with changes in gastrointestinal motility and impairs the proper absorption of intestinal nutrients [54]. In order to confirm that forced treadmill running was stressful to exercising animals, Sasaki et al. [57] reported that the serum levels of corticosterone and norepinephrine (NE) in the kidney and liver were significantly higher in the treadmill group as compared to the levels of these hormones in the voluntary wheel-running group of mice, thus indicating that treadmill exercise was rather a stressful condition. 

It is well known that strenuous exercise leads to oxidative stress and that the reactive oxygen species (ROS) can modulate the activity of pathways involved in the production of pro-inflammatory mediators [55,56]. The oxidative stress caused by intensive exercise may also play role in the etiology of the compromised intestinal barrier integrity by disrupting tight junctions and decreasing viability of epithelial cells [57,58,59,60]. It has been demonstrated that exercise induces oxidative stress but, in addition, the increased antioxidant enzyme activities have been observed after exercise considered as stressor [57,61,62,63,64]. In line with these reports, we presented the evidence that the mRNA expression for antioxidative enzymes SOD-1, SOD-2, and GPx but not HO-1 mRNA was markedly increased in force exercising mice with colitis as compared with the mRNA expression of SOD-1, SOD-2, and GPx in sedentary mice with colitis. This could be explained by the compensatory defensive response of intestinal mucosa exposed to oxidative stress resulting from the inflammatory reaction and forceful conditions of treadmill exercise.

In conclusion, the moderate exercise appears to be safe and has been encouraged for IBD patients as beneficiary in the prevention of disease relapse, the maintenance of nutritional status and improvement of quality of life [65]. 

## 5. Conclusions

The moderate exercise appears to be safe and has been encouraged for IBD patients as beneficiary in the prevention of disease relapse, the maintenance of nutritional status and improvement of quality of life [65]. However, our present study supports and further extends previous observations that the prolonged and intensive exercise could lead to escalation of inflammatory responses as manifested by the increase in the lipid peroxidation, as well as gene and protein expression of HIF-1α and release of inflammatory markers, finally leading to exacerbation of colitis and marked impairment of the healing of these inflammatory changes in colonic mucosa of obese and non-obese animals. These pathological changes could be caused, at least in part, by a fall in colonic microcirculation and an increase in oxidative stress. It is not excluded that the high fat diet which leads to obesity acts locally via an accumulation of mesenteric fat in abdominal cavity indicating that this pathologically modified adipose tissue could also contribute to the increased severity of experimental colitis observed in our present study.

## Figures and Tables

**Table 1:** The nucleotide sequences of the primers used in real time PCR.

Gene	Forward Primer (5′-3′)	Reverse Primer (5′-3′)
COX-2	GCCAGCAAAGCCTAGAGCAACAAA	TACTGAGTACCAGGCCAGCACAAA
GPx-1	ACAGTCCACCGTGTATGCCTTC	CTCTTCATTCTTGCCATTCTCCTG
HO-1	CACGCATATACCCGCTACCT	CCAGAGTGTTCATTCGAGCA
IL-1β	TGGAGAGTGTGGATCCCAAGCAAT	TGCTTGTGAGGTGCTGATGTACCA
IL-6	TTGTACAGTCCCAGTCAGGCAACA	TCAAGCTACTGCAGGCCAGTTACA
iNOS	CAAACACGAGTGCAGCTGGTTGAA	AGGCAGGACTGAGTTCAGTGTGTT
SOD-1	CCACGTCCATCAGTATGGGG	CGTCCTTTCCAGCAGTCACA
SOD-2	GTGTCTGTGGGAGTCCAAGG	CCCCAGTCATAGTGCTGCAA
TNF-α	TGAGTTCTGCAAAGGGAGAGTGGT	TGCACCTCAGGGAAGAATCTGGAA
β-actin	CCCATCTATGAGGGTTACGC	TTTAATGTCACGCACGATTTC
